# Evaluation of intravenous regional perfusion with amphotericin B and dimethylsulfoxide to treat horses for pythiosis of a limb

**DOI:** 10.1186/s12917-015-0472-z

**Published:** 2015-07-16

**Authors:** Renata GS Dória, Mariana B Carvalho, Silvio H Freitas, Luciane M Laskoski, Edson M Colodel, Fábio S Mendonça, Marco AG Silva, Renan Grigoletto, Paulo Fantinato Neto

**Affiliations:** Department of Veterinary Medicine, University of São Paulo, Duque de Caxias Norte ave 225, Pirassununga, ZIP 13635-900 SP Brazil; Department of Veterinary Medicine, University of Cuiabá, Cuiabá, MT Brazil; Department of Veterinary Medicine, Federal University of Paraná, Curitiba, PR Brazil; Department of Veterinary Pathology, Federal University of Mato Grosso, Cuiabá, MT Brazil; Department of Morphology and Physiology, Federal Rural University of Pernambuco, Recife, PE Brazil; Department of Veterinary Medicine, Federal University of Tocantins, Araguaína, TO Brazil

**Keywords:** Amphotericin B, Dimethylsulfoxide, Horse pythiosis, Intravenous regional perfusion, Lesion healing

## Abstract

**Background:**

Treatment for horses with pythiosis of a limb is challenging. This study aims to evaluate the effects of administering amphotericin B in a 10 % solution of dimethylsulfoxide by intravenous regional limb perfusion (IRLP) to treat horses for cutaneous pythiosis of a limb.

**Results:**

All 15 of the horses treated had complete resolutions of their lesion between 6 to 9 weeks after a single IRLP treatment. No complications were observed at the site of venipuncture for IRLP. Before initiation of treatment, there was anemia and marked leucocytosis which resolved following treatment. Serum biochemistry showed no significant changes.

**Conclusions:**

IRLP administration of amphotericin B in a 10 % DMSO solution was easily performed, relatively inexpensive and an effective treatment for treating horses for pythiosis of a limb and resolved the infection with no complications.

## Background

Treatment of horses for infection of a limb with *Pythium insidiosum* is challenging, and its effectiveness appears to depend on such factors as size and site of the lesion, duration of infection, immunocompetence, and the type of treatment. Systemically administered antifungal drugs, such as potassium or sodium iodide, ketoconazole, miconazole, fluconazole, itraconazole, and amphotericin B, have been administered with or without the surgical excision of the lesion to improve outcomes [1–3]. However, these drugs are considered hazardous and are expensive when administered systemically to horses, and because *Pythium insidiosum* is not a true fungus, this protistal organism has increased resistance to most available antifungal agents [3, 4]. Dória et al. [5] reported that intravenous regional limb perfusion (IRLP) with amphotericin B is effective for treating horses with a cutaneous lesion of pythiosis of a limb, resolving the infection with manageable complications, such as oedema of the limb, signs of pain during palpation of the limb, and inflammation at the site of venipuncture for IRLP. Their study demonstrated that 92 % of the horses (*n =* 11) affected by *Pythium insidiosum* and presenting with exuberant granulation tissue in the distal aspect of a thoracic or pelvic limb that were treated with intravenous regional perfusion with amphotericin B had complete resolution of their lesions 35 days after one treatment or 60 days after two treatments. Dória et al. [5] considered the intravenous regional perfusion of amphotericin B to be an effective adjunct therapy to surgical excision and thermocauterization in treating horses for pythiosis of the distal portion of the limb.

We hypothesized that better results with fewer complications might be achieved if dimethylsulfoxide (DMSO) is added to the perfusate when amphotericin B is administered by IRLP as a treatment for pythiosis of a limb. We reasoned that adding DMSO to the perfusate would achieve a higher concentration of amphotericin B within the infected tissues, and because DMSO has been shown to be an effective anti-inflammatory agent, its addition to the perfusate would reduce inflammation at the site of intravenous administration. It may also possess some inhibitory effects on the growth of a variety of fungi as a result of its effect on the immune response and as a result of reducing endotoxin-induced tissue damage. Studies have shown that intravenous administration of DMSO results in vascular dilation and increased flow of blood through experimentally created cutaneous flaps [6–9]. Such properties associated with its ability to penetrate biological membranes provide a rationale for its use in conjunction with an antifungal drug for regional limb perfusion [10].

Thus, we evaluated the effects of administering amphotericin B in a 10 % solution of DMSO by IRLP as an adjunct therapy to surgical excision, hoping to diminish the detrimental vascular effects associated with administration of amphotericin B, to treat horses for pythiosis of a limb.

## Methods

This study was approved by the University of Cuiabá Animal Care and Ethics Committee, under protocol number 2009–228. Fifteen horses (8 males and 7 females, age 4 months to 15 years, weighing 100–420 kg) diagnosed with pythiosis were studied. This research was carried out on horse farms located in Tocantins state (*n* = 1), São Paulo state (*n =* 2) and Mato Grosso state (*n =* 12), Brazil. The presumptive diagnoses were made on the basis of historical data, the gross appearance of the granulomatous lesion, and the histological appearance of the lesion. Lesions were located distal to the cubital joint (elbow) or the femorotibial joint (stifle). In preparation for the treatment, feed was withheld for 12 h before treatment was administered. A catheter was placed into a jugular vein, and the horses were tranquillized with acepromazine (0.1 mg/kg) and then anesthetized with guaiacol glycerol ether (100 mg/kg), ketamine (2 mg/kg), and midazolam (0.1 mg/kg). Horses were positioned in lateral recumbency with the lesion uppermost. The site of the lesion was prepared for surgery by scrubbing the site with soap and applying dilute povidone-iodine solution and 70 % isopropyl alcohol. An Esmarch tourniquet was applied proximal to the lesion to prevent hemorrhaging, making it possible to surgically remove the exuberant granulation tissue and kunkers safely avoiding exposing the bone or entering a synovial structure. Tissues (1 cm^3^) were collected and fixed in 10 % formalin for histological (hematoxylin and eosin; Grocott’s methenamine silver) and immunohistochemical (the labeled streptavidin biotin method) analyses, performed as previously reported [11–13]. After surgery, the Esmarch tourniquet was partially released to identify bleeding vessels for cauterization with electric thermocautery, and the site of the lesion was bandaged. The Esmarch tourniquet was repositioned and retightened, and a superficial vein (the cephalic, palmar digital, saphenous, plantar digital, or dorsal digital vein) next to the lesion and distal to the tourniquet was catheterized using a 20-, 22-, or 24-ga catheter for administration of the drugs. The catheterized vein was injected with 60 mL of a solution containing 50 mg (10 mL) of amphotericin B^1^, 6 mL of medical grade DMSO^2^ and 44 mL of lactated Ringer’s solution. The injection was delivered using finger pressure over 5 min using a 60-mL syringe connected to an extension line. The catheter was removed after the administration, and firm pressure was applied manually to the site of venipuncture. The tourniquet was released 45 min after the administration of the solution of amphotericin B and DMSO.

Lesions were evaluated before treatment (Day 0) and then weekly (1 to 9 weeks) after the administration of the solution of amphotericin B and DMSO until the lesions were completely healed. At the same time points, blood samples were collected for haematological and biochemical analyses using standard techniques [14].

The lesion’s size (length and width) was determined at day 0 using a ruler. The lesions were assessed qualitatively, and the response to therapy was determined by clinical observation (i.e., gross appearance of the lesion). The appearance of the lesion was compared to the photographic appearance of the lesion at the times of previous evaluations. A wound was considered completely healed when it was completely covered with epithelium. The affected limb was evaluated for regional swelling, sensitivity to palpation, and signs of inflammation at the site of venipuncture.

The data were analyzed with the Statistical Analysis System (SAS.2011). Normality of the data distribution was evaluated by the Shapiro-Wilk test. A one-way analysis of variance with repeated measures, followed by a Tukey test, was used for the comparisons of the weekly data. The differences were considered to be statistically significant when *P* ≤ 0.05.

## Results

The pythiosis lesions were located in different regions, including the radius (20 %), carpus (6.66 %), metacarpus (6.66 %), tibia (6.66 %), tarsus (6.66 %), metatarsus (6.66 %), fetlock and pastern (46.7 %). At day 0, all of the horses had large lesions ranging from 12 to 50 cm in length (mean length 27 cm) and 15 to 40 cm in width (mean width 25 cm) and surrounded 90° (4 horses), 180° (7 horses) or 360° (4 horses) of the affected areas. The lapse of time between emergence of the lesion and the beginning of treatment with amphotericin B and DMSO ranged from two to five months (average, three months).

Before treatment (D0), the affected limb was swollen, and the lesion was filled with exuberant, ulcerated granulation tissue and surrounded by oedematous tissue. The surface of the granulation tissue was nodular and covered with a mucosanguineous viscous exudate. The tissue sections had sinuses containing kunkers, usually surrounded by seropurulent discharge. The horses showed signs of intense pruritus that was characterized by self-mutilation.

**Fig. 1:**
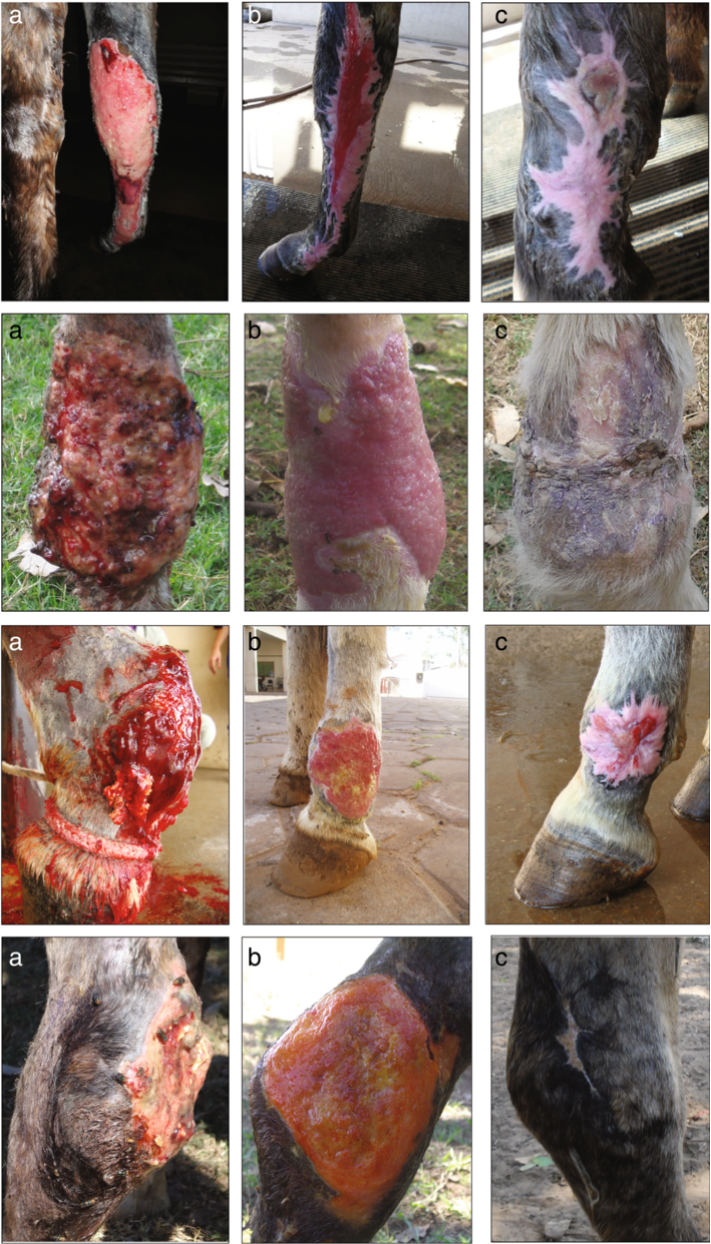
Healing of pythiosis lesions treated with amphotericin B and DMSO by IRLP in horses. Progression of healing of pythiosis lesions in the limb of horses treated with a single administration of amphotericin B in a 10 % DMSO solution by intravenous regional perfusion. Note lesions before (Day 0; left; **a**), 4 (middle; **b**), and 8 (right; **c**)weeks after the treatment

The histopathological diagnosis was pyogranulomatous dermatitis associated with the pseudo hyphae characteristic of *Pythium insidiosum* (subcutaneous pythiosis), confirmed by the immunohistochemistry analysis. Surgical excision of the exuberant granulation tissue and IRLP administration of amphotericin B and DMSO resulted in complete resolution of the lesions. All 15 lesions regressed after a single administration of a solution containing amphotericin B and DMSO. By one week after a single treatment, exudate, sinuses, and kunkers had disappeared from the lesions, and the granulation tissue had regressed and turned from white to pink-yellow. In subsequent weeks, the granulation tissue became pink and flat, and a margin of epithelialization could be seen moving centripetally. Epithelialization was complete by 6 weeks in 5 horses, 4 of which had lesions surrounding 90° of the affected area and 1 of which surrounded 180° of the affected area; 8 weeks in 5 horses of which had lesions encompassing 180° of the limb; and 9 weeks in 5 horses, 1 of which had lesions encompassing 180° of the limb and 4 of which encompassed 360° of the limb (Fig. 1). No complications at the site of venipuncture for RLP were observed in any of the horses, and no horse had recurrence of pythiosis at the time of follow-up, one year after resolution of disease.

Haematology showed that there was an increase in the RBC and PCV after treatment. Anemia resolved by 2 weeks after IRLP. The total and differential leucocyte counts were significantly increased before treatment, with neutrophilia and eosinophilia. The neutrophil count and the eosinophil count decreased and returned to normal values by 2 weeks after treatment. Increased serum concentration of fibrinogen found when the horses were admitted had decreased to normal values by 2 weeks after treatment. No significant abnormalities were noted during examination of the results of other biochemical assays (Table 1).

**Table 1:** Haematology and biochemistry in horses with pythiosis treated by IRLP with amphotericin B and DMSO. Mean values of haematology and selected blood biochemistry tests before (week 0) and after (1 to 9 weeks) intravenous regional perfusion with amphotericin B in a 10 % DMSO solution in 15 horses with pythiosis

	Haematology					Biochemistry						
Weeks	RBC (x10^6^/μL)	PCV* (%)	TLC* (x10^3^/μL)	SN* (x10^3^/μL)	E* (/μL)	Fibrinogen (g/L)	TP (g/L)	AST (UI/L)	GGT (UI/L)	ALP (UI/L)	CR* (μmol/L)	U (mmol/L)
0	6.17	26^ab^	21^a^	15.38^a^	949.83^a^	5.2	74^d^	192^d^	17.30^d^	306.53^d^	112.27^ad^	13.24^d^
1	5.54	24^a^	14.32^ab^	10.15^ab^	827.20^ab^	4.6	75^d^	163^d^	20.78^d^	213.33^d^	74.26^bd^	13.02^d^
2	6.65^d^	28^ab^	11.54^bcd^	7.41^bcd^	577.43^abd^	4^d^	77^d^	167^d^	18.49^d^	254.12^d^	79.56^bd^	14.40^d^
3	6.74^d^	29^ab^	10.77^bcd^	6.24^bcd^	571.83^abd^	3.7^d^	75^d^	181^d^	15.38^d^	222.53^d^	79.56^bd^	13.97^d^
4	7.25^d^	31^b^	10.48^bcd^	5.98^bcd^	438.33^abd^	2^d^	75^d^	194^d^	13.77^d^	273.51^d^	78.68^bd^	16.25^d^
5	7.45^d^	31^b^	9.28^bcd^	5.32^bcd^	346.67^abd^	2.7^d^	72^d^	214^d^	15.94^d^	268.19^d^	80.44^bd^	17.04^d^
6	7.34^d^	32^bd^	8.67^bcd^	4.55^cd^	334.17^abd^	3^d^	72^d^	184^d^	17.85^d^	210.73^d^	80.44^bd^	18.58^d^
7	7.38^d^	31^b^	8.38^cd^	5.04^bcd^	287.17^bd^	2^d^	72^d^	196^d^	14.17^d^	211.73^d^	94.59^abd^	17.83^d^
8	7.80^d^	33^bd^	9.13^bcd^	5.06^bcd^	315^abd^	2^d^	72^d^	195^d^	14.40^d^	200.18^d^	84.86^abd^	13.54^d^
9	7.66^d^	32^abd^	9.13^bcd^	5.41^bcd^	189^abd^	2^d^	69^d^	221^d^	13.80^d^	277.28^d^	83.98^abd^	13.05^d^
Reference range^22^	6.4–10.0	32–47	5.2–13.9	2.26–8.58	0–600	1–4	57–79	152–294	9–25	143–395	88.4–167.96	9.21–19.96

*RBC* red blood cell count, *PCV* packed cell volume, *TLC* total leukocytic count, *SN* segmented neutrophils, *E* eosinophils, *F* fibrinogen, *TP* total protein, *AST* aspartate aminotransferase, *GGT* gamma-glutamyltransferase, *ALP* alkaline phosphatase, *CR* creatinine, *U* urea nitrogen

*Significant at probability ≤ 0.05

^a^; ^b^; ^c^ - means with different letters are significantly different (Tukey’s test; *P* ≤ 0.05); d Mean value inside reference range

## Discussion

We report the successful treatment of horses for cutaneous pythiosis of the limb after IRLP using amphotericin B and DMSO in combination with excision of the exuberant granulation tissue. Our results indicate that the treatment we administered is successful, cost-effective, and easily performed.

The challenge of treating pythiosis is characterized by the severity of the disease and by the absence of gold standard chemotherapy. Complete surgical excision is the treatment of choice, but the disease is often too extensive at the time of diagnosis to allow complete resection. Immunotherapy and/or the systemic, intralesional, and topical administration of antifungal drugs has been combined with surgical excision to improve therapeutic efficacy [3, 15]. Amphotericin B, when administered systemically, does not reach a therapeutically effective concentration at the site of infection with *Pythium insidiosum* and can be associated with adverse effects such as nephrotoxicity and anaemia [16, 17]. However, IRLP has been shown to be an effective technique for obtaining high drug concentrations in tissue [18–21], and the high concentration of amphotericin B in the infected tissue enhances the efficacy of the drug against *Pythium insidiosum*. Increased concentrations of amphotericin B impair or inhibit this oomycete’s essential metabolic processes and result in the death of the microorganism [5], as demonstrated in this study.

Systemically administered medical treatment of horses for pythiosis has been reported to be marginally effective when the lesions were located in the limbs [22]. Local thrombosis, ischemia, tissue necrosis, and abscess formation, the typical characteristics of pythiosis lesions, decrease the local blood supply and thus decrease the effective therapeutic drug concentration that reaches *Pythium insidiosum* [23, 24]. The inability to completely resect a lesion because of its proximity to joints or bones adds to the challenge of treating horses for cutaneous pythiosis and favours the persistence of the infection. According to some previous reports, only horses with small, superficial lesions of pythiosis can be treated successfully [25, 26]. Dória et al. [5], however, were able to resolve the lesion caused by pythiosis in 92 % of affected horses, regardless of the size of the lesion, by using a combination of either complete or partial surgical excision, thermocautery, and administration of one or two IRLP of amphotericin B. Complications associated with IRLP of amphotericin B, such as oedema of the limb, signs of pain during palpation of the limb, and inflammation at the site of venipuncture, were local and resolved. Similarly, the current study demonstrates that the combination of amphotericin B and DMSO provides excellent results. All 15 of the horses (100 %) showed complete resolution of their lesion after a single intravenous regional perfusion, without systemic or local complications, and all lesions were large, some involving the complete circumference of the limb.

Dória et al. [5] reported that excision of the exuberant granulation tissue alone is not sufficient to resolve an infection of a limb caused by *Pythium insidiosum*. In chronic conditions, there is more fibrosis and scarring, which further isolate the protistal organism from the body’s defense mechanisms as well as from systemically administered antifungal drugs [24, 27]. A high concentration of an antifungal drug can be achieved in chronically infected tissue by intravenous regional perfusion [24, 28], as demonstrated in this study. All of the horses treated with IRLP of amphotericin B in a 10 % DMSO solution had complete resolution of their lesion with no recurrence at the time of follow-up one year later, even though excision of the infected tissue was incomplete.

We observed, 1 week after treatment, that the granulation tissue was devoid of exudate and that the sinuses and kunkers were no longer present, indicating that infection by *Pythium insidiosum* had resolved completely. Healing period was related to the size of the lesion. The larger the lesion, the longer the time the lesion took to heal.

The horses had anaemia before surgery and this has been observed previously [29–31]. There was also marked leukocytosis with neutrophilia and eosinophilia before treatment [2, 16]. Administration of a combination of amphotericin B and DMSO by IRLP was effective in treating horses for cutaneous pythiosis of the limb and evidence of resolution of infection included a marked decrease in the total leukocyte count by 2 weeks and return of other haematological values, such as plasma concentrations of acute phase protein and fibrinogen, to normal by 2 weeks after treatment. No changes were observed in the serum enzyme activities after the IRLP of amphotericin B and DMSO suggesting that IRLP with amphotericn B did not cause systemic complications, such as liver and/or kidney dysfunction, which have been reported to occur when amphotericin B is administered systemically [32–35]. Studies have found that by administering a drug by IRLP, the concentration of the drug at the site of the disease can be increased many fold without risk of reaching a toxic concentration of the drug systemically [19, 24, 36–38]. IRLP allows a wider use of drugs that are considered hazardous and expensive when systemically administered to horses [39, 40], such as amphotericin B [4, 17, 34, 35, 41].

We administered 50 mg amphotericin B in lactated Ringer’s solution by IRLP, as reported by Dória et al. [5] but added 6 mL DMSO (10 % of the total volume) to compose the final solution. The dose and the total volume of amphotericin B (0.83 mg/mL/60 mL) and DMSO (6 mL/60 mL) were standardized in this study and were administered irrespective of the horse’s age and body weight, the size of the lesion, and the site on the limb at which the tourniquet was applied. We believe that the addition of DMSO to the perfusate prevented vascular inflammation typically induced by IRLP of amphotericin B and reported by Dória et al. [5]. DMSO is a potent anti-inflammatory agent that acts, through the activity of superoxide dismutase, to suppress production of prostaglandins, to limit production of free radicals, and to scavenge free radicals [9, 42]. DMSO has multiple properties that can be beneficial when used as adjunctive treatment for sepsis. DMSO reduces platelet aggregation and thus decreases the incidence of thrombus formation in small vessels. This activity helps to normalize tissue perfusion in the face of the vascular insults that often accompany septic conditions of the equine extremities. The inhibitory effects of DMSO on the growth of a variety of bacteria, viruses, and fungi have been documented by a number of studies [6, 7]. The bacteriostatic and bactericidal effects of DMSO may be the result of the drug’s ability to penetrate biological membranes [8, 9]. A drug that possesses these qualities is likely to attenuate some of the deleterious effects of vascular injury and free radical production inherent in septic conditions of the limb by affording a potent anti-inflammatory effect and aiding in tissue perfusion [10]. It is noteworthy that DMSO is not a licensed drug therefore it is not allowed for treatment in many countries.

We assumed that by using an appropriate rate of infusion, the increased intravascular pressure would favour the diffusion of amphotericin B through the tissues and enhance the drug’s concentration in the target tissues [40, 43, 44] and that the combination with DMSO would increase the drug’s diffusion through the affected tissues, providing better resolution of infection than when amphotericin B is administered alone. In contrast with the study by Dória et al. [5], here only one IRLP with amphotericin B and DMSO was necessary to resolve a large wound on a limb caused by chronic pythiosis. Administering DMSO and a low dosage of amphotericin B by IRLP caused no systemic or local complications and is likely to be safer and more economical than administering amphotericin B systemically.

## Conclusion

This small case series demonstrates that in association with surgical excision of the exuberant granulation tissue caused by *Pythium insidiosum* infection, a single IRLP administration of 50 mg of amphotericin B and DMSO (10 % of the total solution) was an effective technique for treating horses for pythiosis of a limb that produced no noticeable local or systemic complications.

## Abbreviations

IRLP: Intravenous regional limb perfusion
DMSO: Dimethylsulfoxide
RBC: Red blood cells
PCV: Packed cell volume

## Footnotes

1 Anfotericin B®, Cristália, São Paulo, Brazil.

2 Dimesol®, Marcolab, São Paulo, Brazil.
